# San Diego Thornmint (*Acanthomintha ilicifolia*) Populations Differ in Growth and Reproductive Responses to Differential Water Availability: Evidence from a Common Garden Experiment

**DOI:** 10.3390/plants12193439

**Published:** 2023-09-29

**Authors:** Katherine D. Heineman, Stacy M. Anderson, Joseph M. Davitt, Laurie Lippitt, Bryan A. Endress, Christa M. Horn

**Affiliations:** 1 Center for Plant Conservation, 15600 San Pasqual Valley Rd., Escondido, CA 92027, USA; kheineman@saveplants.org; 2 San Diego Zoo Wildlife Alliance, 15600 San Pasqual Valley Rd., Escondido, CA 92027, USA; sanderson@sdzwa.org (S.M.A.); jdavitt@sdzwa.org (J.M.D.); lauriel@infostations.com (L.L.); 3 Eastern Oregon Agriculture Research Center, Oregon State University, 372 S. 10th Street, Union, OR 97883, USA; bryan.endress@oregonstate.edu

**Keywords:** annual plants, common garden experiment, drought responses, endangered species, germination, threatened species, viability testing

## Abstract

The responses of rare plants to environmental stressors will determine their potential to adapt to a rapidly changing climate. We used a common garden approach to evaluate how six populations of the annual San Diego thornmint (*Acanthomintha ilicifolia* Lamiaceae; listed as endangered in the state of California and as threatened by the US Fish and Wildlife Service) from across the species range respond in terms of growth (biomass, height, and width) and reproduction (seed production, floral production, and next generation seed viability) to experimental differences in water availability. We found a significant irrigation-by-population interaction on the aboveground growth, wherein the differences in the magnitude and direction of treatment did not correlate directly with climate variables in natural populations. With respect to reproduction, the low-irrigation treatment produced more seeds per plant, more reproductive individuals, and a larger proportion of viable seed in most, but not all, populations. The seed production and the effect of irrigation on seed production correlated positively with rainfall at wild source populations. These results suggest that *Acanthomintha ilicifolia* responds to water limitation by creating more and higher-quality seed, and that plants locally adapted to a higher annual rainfall show a greater plasticity to differences in water availability than plants adapted to a lower annual rainfall, a finding that can inform the in situ demographic management and ex situ collection strategy for *Acanthomintha ilicifolia* and other rare California annuals.

## 1. Introduction

Currently, nearly 40% of the world’s plants are threatened with extinction [1], and climate change is increasingly being recognized as a threat to these plants. Rare species are disproportionately threatened by climate change, as well as other human impacts [2,3,4,5]. Thus, predicting how rare and threatened plants will react to climate change is a high and important priority for the conservation of species and the ecosystems that they occupy.

There are three possible outcomes for plant populations in a rapidly changing climate: in situ adaptation, migration, or extirpation [6,7]. Increased knowledge about the species, especially in terms of their ecological needs and their adaptive potential, can help us to identify appropriate conservation strategies in the face of climate change. Different constraints on species, including their phenotypic plasticity and adaptive potential, impact which of the three outcomes is most likely, and what strategies to undertake: assisted migration, the management of biodiversity corridors, the augmentation of the potential climate, the protection of genetic refugia, and/or ex situ conservation [7].

Ex situ conservation, largely in the form of conservation seed banking, plays a supporting role in many of these strategies, in addition to serving as a long-term safeguard against loss of genetic diversity due to extirpation and extinction [8]. There is an increasing interest in being more strategic about conservation seed collecting [9,10], and knowing more about potential local adaptation can help inform such strategies. Beyond long-term ex situ conservation, the use of the collected seed as a source for restoration efforts should consider the potential of genetic differences between populations and the potential of both locally adapted traits and phenotypic plasticity [11,12].

Common garden experiments help researchers and conservationists plan safeguards for a species, both in situ and ex situ, by examining how the local environment is driving the expression of intraspecific variation, how species or populations respond to changing climatic conditions, and more [13,14,15,16]. Many studies examining species population or species responses to climate change have established common gardens with different water availability, sometimes in conjunction with other variables, such as warming or competition [15,17]. However, the impact of drought on resource allocation for plants, specifically annuals, is not always predictable [18]. Nonetheless, common garden experiments are useful tools for gauging species- and population-level responses to environmental stress in species of a high conservation value, such as the San Diego thornmint (*Acanthomintha ilicifolia* (A.Gray) A.Gray, Lamiaceae), an herbaceous annual.

Responses to climate change may be more important to the long-term persistence of edaphic specialist plant species than to that of other rare plants, as migration to a more suitable habitat is limited by the availability of the edaphic habitat [19]. In California, climate change is predicted to shrink the edaphic habitat of rare annual herbs specialized to the hydrological and edaphic environment of vernal pools and similar habitats [20]. *Acanthomintha ilicifolia* is one such annual—a mint that is listed as endangered in the state of California and as threatened by the US Fish and Wildlife service due to the rapid loss of its native clay lens habitat spanning San Diego County, to northwest Baja California, Mexico [21]. Although an edaphic specialist, *A. ilicifolia* spans relatively broad elevation and precipitation gradients. *A. ilicifolia* produces bisexual flowers and is an outcrosser that is insect pollinated; however, there is limited information regarding its breeding system. A genetic analysis of 21 *A. ilicifolia* populations found a strong genetic structure among populations and at least two cytotypes [22]. A more recent genomic study found that these populations make up at least five unique genetic clusters within San Diego County [23], making *A. ilicifolia* a promising study species for examining the effects of local adaptation on genetic and phenotypic diversity in rare plant species.

In this study, we evaluate how experimental variation in water availability affects aspects of the growth and reproductive output of *A. ilicifolia* relevant to its long-term persistence in the wild and in ex situ conservation. We used a common garden approach to test the interactive effects of source population and irrigation treatments on the aboveground growth (biomass, height, and width) and reproductive output (flower number, seed number, and seed viability) of six populations of *A. ilicifolia* spanning a regional precipitation gradient. We hypothesized that, if plants allocate more resources to structures supporting light and root competition when water is an abundant resource, then plants supplied with ample water will invest proportionally more in aboveground growth and less in reproductive output compared to plants grown under drought stress. We further hypothesized that plants grown from seed sourced from populations with a lower average annual precipitation in nature should perform relatively better in the low-water treatment than populations with a higher average annual precipitation. Uncovering how threatened and endangered annual plants vary in key fitness traits across time and space is critical to developing an informed conservation strategy. 

## 2. Results

### 2.1. Common Garden Experiment

**Figure 1:**
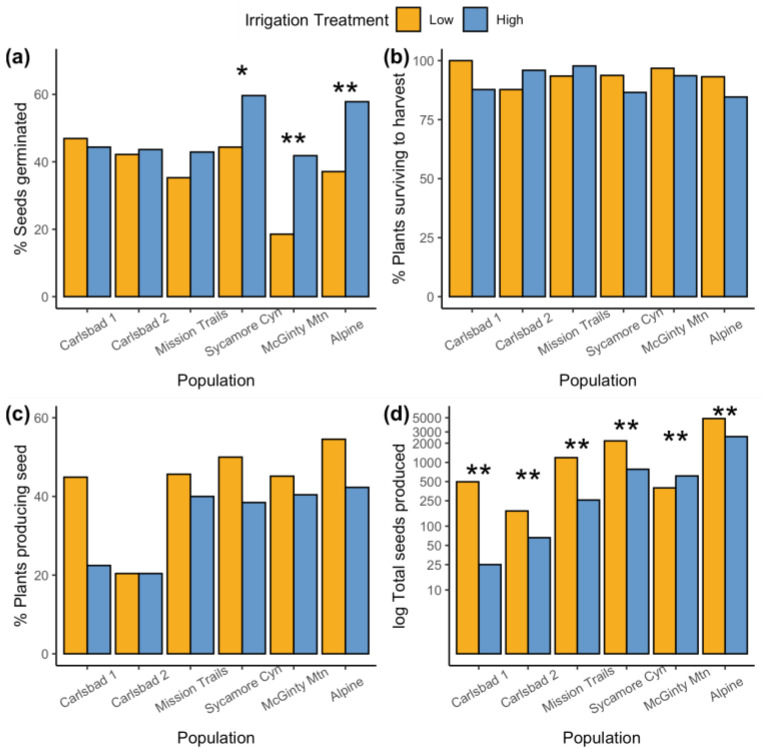
Comparison of the pot-based germination and reproductive performance between treatments for each population in the common garden experiment, including (**a**) the percentage of wild collected seed that germinated in pots, (**b**) the percentage of plants that survived to harvest, (**c**) the percentage of plants that produced one or more seeds, and (**d**) the total seed produced across all plants. Stars above population-by-treatment pairs (* and **) represent significant differences at alpha = 0.05 and 0.001, respectively, between treatment groups, determined via post hoc contrasts of generalized linear models evaluating the interaction effect of populations and treatments on the binomial and count-based metrics of seed performance. The populations are ordered on the x-axis by increasing mean annual rainfall.

The response of *Acanthomintha ilicifolia* to experimental watering treatments differed among source populations at all stages of plant growth and reproduction. The germination rate of wild collected seeds planted in common garden pots was 42% across all the source populations and treatments. We observed a significant treatment-by-population interaction on the germination success (X^2^ = 35.2, *p* < 0.001), wherein the high-irrigation treatment had a greater germination success than the low-irrigation treatment in three of the six source populations, but this did not differ significantly between treatments in the two Carlsbad populations or the Mission Trails population (Figure 1a). One treatment in particular, the low-irrigation treatment for the McGinty Mountain population, showed only 20% germination and did not yield a single germinant in 24 of the 55 experimental pots. After thinning, 91% of the plants survived to harvest, and a population-by-irrigation interaction on survival (X^2^ = 15.4, *p* = 0.009) was primarily driven by a higher proportion of surviving individuals in the low-irrigation treatment compared to the high-irrigation treatment in the Alpine and Carlsbad North 1 populations, whereas the reverse trend was true for the Carlsbad North 2 population (Figure 1b).

**Figure 2:**
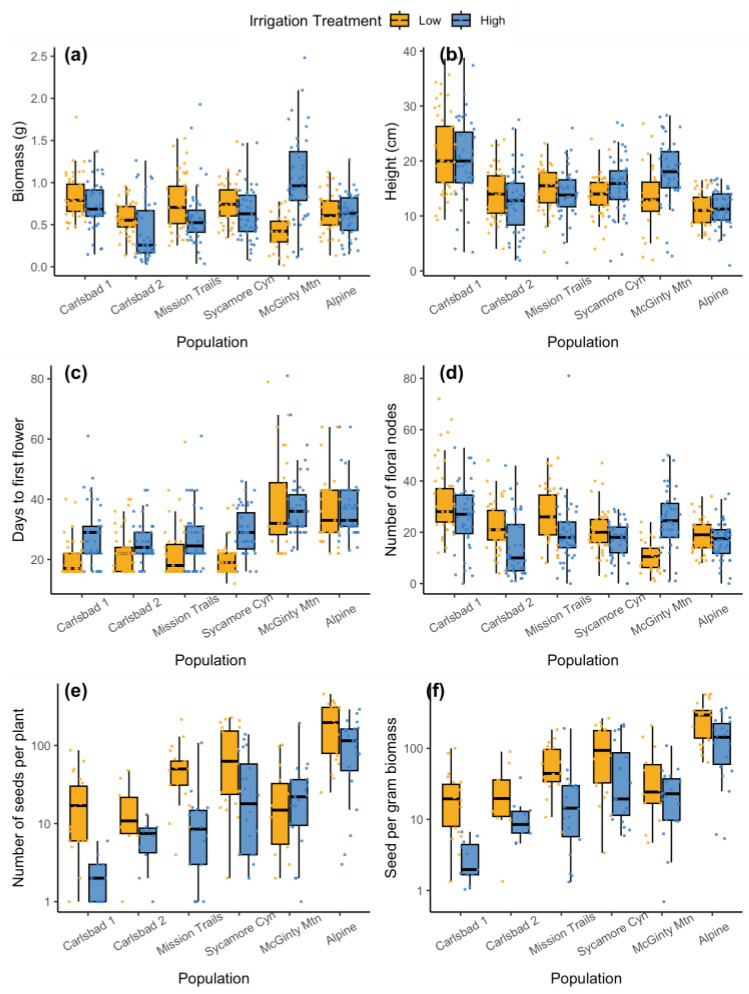
Box plots comparing individual plant measures of performance in the common garden experiment among populations and treatments, as measured when the experiment plants were harvested, including (**a**) aboveground biomass, (**b**) height, (**c**) width, (**d**) number of floral nodes, (**e**) number of seeds per plant, and (**f**) seeds per gram biomass (only for plants producing seeds). The populations are ordered on the x-axis by increasing mean annual rainfall.

The source population explained a greater proportion of variance than the irrigation in the average plant allocation to aboveground metrics such as biomass, height, width, flower number, and seed production (see *X*^2^ values, Table A1). In general, the Carlsbad 1 population produced the largest plants, and the Alpine population produced the smallest plants. All the linear models evaluating aboveground allocation had a significant population-by-treatment interaction due to the response of the southernmost McGinty Mountain source population, which had taller and wider plants, with a higher biomass and more flowers, in the high-irrigation treatment than in the low-irrigation treatment—the reverse of what was found for the other populations (Figure 2).

The effect of the watering treatment on seed production was more consistent in terms of the qualitative direction of the response across populations. Across all populations and treatments, 42% of plants produced at least one seed. There was a significant main effect of both the population (*X*^2^ = 21.7, *p* < 0.001) and the treatment (*X*^2^ = 6.01, *p* = 0.014) on the probability of seed production. Plants under the low-irrigation treatment were 65% more likely to produce seed than plants under the high-irrigation treatment (Figure 1c). Plants in the low-irrigation treatment also produced more than double the number of seeds on average per plant (Table 1) in all populations except for McGinty Mountain (Figure 2). This resulted in a significant treatment-by-population interaction effect on both the seeds produced per plant (*X*^2^ = 13.0, *p* = 0.024) and the seeds produced per unit of biomass (*X*^2^ = 14.5, *p* = 0.013). Taken together, the higher proportion of seed-producing plants and the higher average seed production per plant resulted in the lower-irrigation treatment having an order-of-magnitude-higher combined seed production compared to the high-irrigation treatment in all populations except McGinty Mountain, for which the reverse was true (Figure 1d). The populations that produced more seed under low-watering treatments also flowered more quickly than plants under the high-watering treatment, but there was no phenological difference among treatments for the McGinty Mountain or Alpine populations (Figure 1c, *X*^2^ = 3.8 *p* = 0.002).

### 2.2. Ex Situ Germination Trials

The germination rates of the wild seed collected in 2013 and stored at room temperature/humidity until 2019 ranged from 85 to 100% (Table A3). The results of more recent viability tests of wild seed collected from these and other *A. ilicifolia* populations tested within a year of collection by the San Diego Zoo Botanical Conservation Center range from 82 to 95%, indicating that very little viability was lost due to storage conditions. However, the ex situ germination rates of the seed produced from the common garden experiment were significantly lower than the wild collected seed in every population.

**Figure 3:**
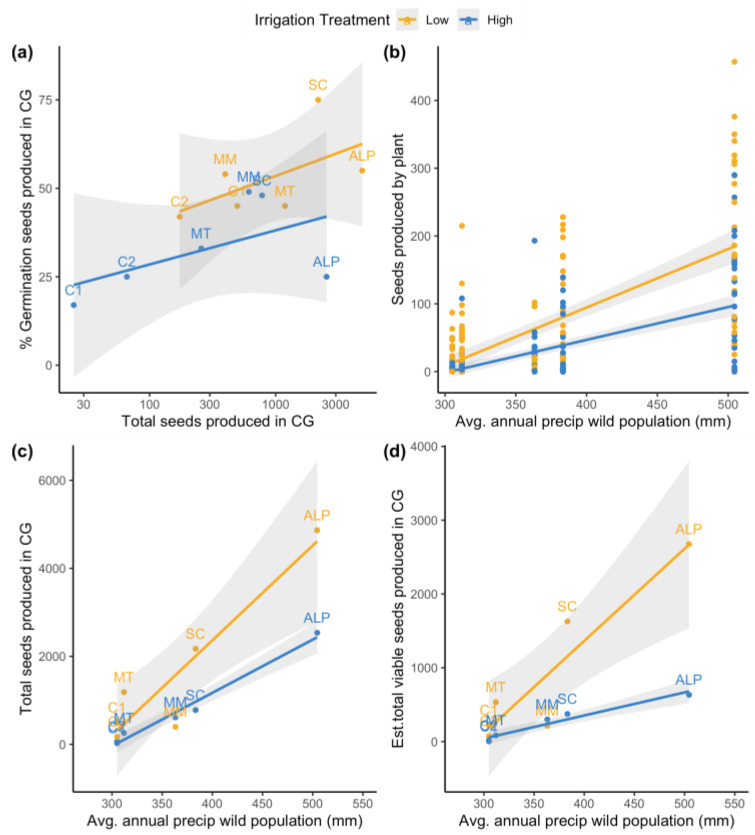
The relationship between (**a**) the seed viability and seed production in each common garden (CG) experiment treatment by population group (see Table 1 for population code definitions), and the relationship between seed production and annual wild source population rainfall, in terms of (**b**) the individual common garden plants, (**c**) the total seeds produced, and (**d**) the estimated viable seeds produced in each treatment by population group. Grey confidence envelopes represent one standard error.

**Figure A1:**
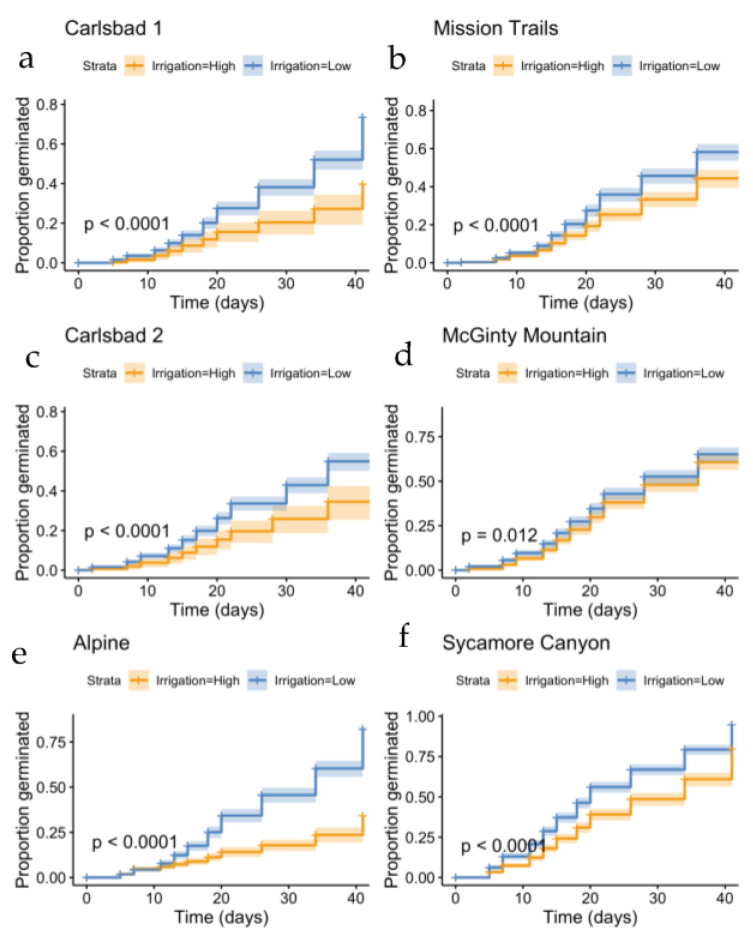
The germination over time (days) by population and treatment in the germination tests on the F1 seed produced from the common garden experiment and tested in 2019 at the San Diego Zoo Botanical Conservation Center, seven years after the common garden experiment. *p* values are associated with Cox proportional hazard regression models testing the difference among treatments for each population.

Nonetheless, we found a significant irrigation-by-population interaction on the probability of germination in the seed harvested from the common garden experiment (likelihood ratio test = 749.5, df = 11, *p* < 0.001; Figure A1). While there was no difference in the germination success among treatments in the McGinty Mountain population (Figure A1d), a significantly higher proportion of the seed harvested under the low-irrigation treatment germinated by the end of the trial compared to seed from the high-irrigation treatment in five of the six populations (Figure A1b). The relationship between seed viability and seed production across the treatment level was positive, but only marginally significant (*X*^2^ = 2.9, *p* = 0.084). However, in five of the six populations, the low-irrigation treatment produced more seeds in total, as well as a higher proportion of viable seed (Figure 3a), indicating that the low-water treatment enhanced both the quantity and quality of the seed produced.

### 2.3. Relationship between Common Garden Reponses and Rainfall in Wild Source Populations

**Figure A2:**
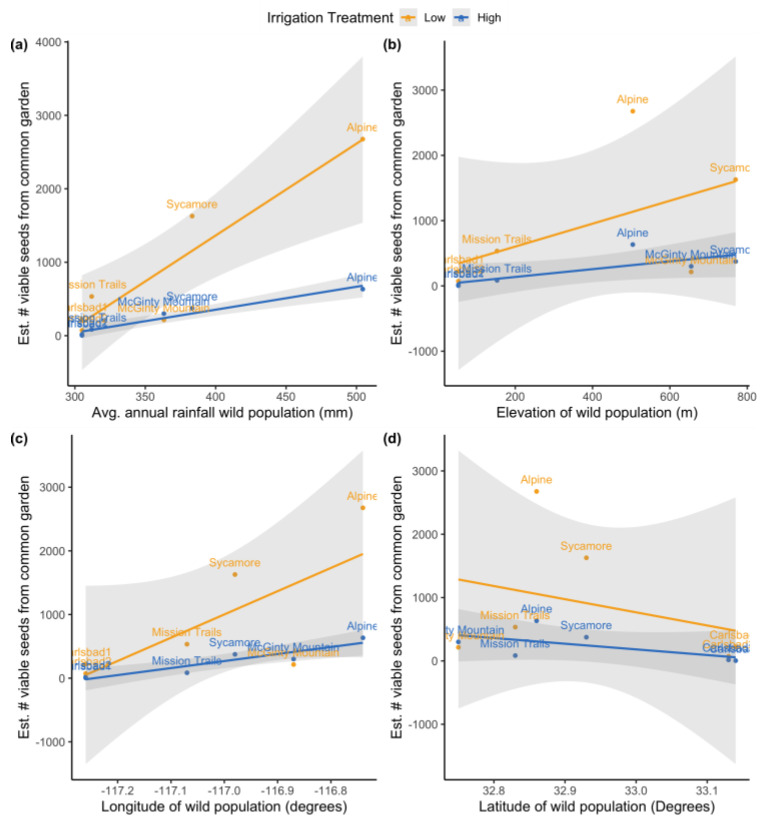
The relationship between the estimated total number of viable seeds produced by each population by treatment group (see Table 1 for population code definitions) in the common garden versus the measure of the source population climate and geography, including (**a**) the average annual rainfall in mm, (**b**) the elevation, (**c**) the longitude, and (**d**) the latitude. Grey confidence envelopes represent one standard error.

The wild population climate, specifically the average annual rainfall, explained a significant proportion of the variation observed among populations in the common garden experiment with respect to the reproductive output, but not the aboveground growth (Table A2, Figure 3). Plants from wetter source populations produced more seed on average than those from drier populations, and a significant treatment-by-population interaction (*X*^2^ = 8.2, *p* = 0.004) suggests that wetter source populations demonstrated a stronger response to this experimental water stress than drier populations (Figure 3b). When the seed totals were aggregated by treatment and population group, this interaction persisted, with the total seed production increasing with the average annual wild population rainfall. The positive effect of low irrigation was more pronounced at the wetter end of the rainfall gradient (*X*^2^ = 6.3, *p* = 0.010, Figure 3c). While the proportion of viable seed produced from the experiment did not scale significantly with the wild source population rain availability (*X*^2^ = 0.45, *p* = 0.509), the estimate of the total viable seeds produced in each treatment by population group increased sharply with the wild population in the low-irrigation treatment, whereas the viable seed production increased more slowly with the rainfall in the high-irrigation treatment (interaction effect, *X*^2^ = 13.0, *p* < 0.001, Figure 3d). Other geographic variables related to the source population (elevation, latitude, longitude) either had no effect, or had a weak effect, on the treatment-wide reproductive output (Figure A2, Table A4) compared to the strong effect of the annual rainfall.

## 3. Discussion

### 3.1. Variable Growth Responses among Acanthomintha ilicifolia Populations in Common Garden

A rare plant with a climatically heterogeneous but edaphically restricted native range, *A. ilicifolia* displays a strong genetic structure among populations and significant differences in growth and reproduction among populations in a common garden setting [22,23]. Our findings add that *A. ilicifolia* populations vary in their responses to experimental water variability and exhibit directional patterns in reproductive performance along a regional climate gradient. The direction of the relationship between the wild population rainfall and common garden reproductive responses was counter to our expectations. We hypothesized that plants adapted to drier environments, where the aboveground competition is lower, should invest proportionally more resources in reproduction and be better able to respond to low water availability than plants from wetter environments. However, we found the opposite to be true. One possible explanation could be that the wet population plants experienced a higher degree of water stress (in both treatments) than the plants from dry environments at our common garden site, which is lower, drier, and warmer than that of the source populations. This relative increase in stress may have triggered wet population plants to invest a greater proportion of resources in reproduction compared to plants adapted to dry populations. Reports of correlations between the source population climate and plant responses, including reproductive phenology [24], physiology [25], and seed and leaf traits [26], have been observed in other plant species and can be interpreted as products of local adaption to climatic conditions, which may be partially responsible for the strong genetic structure among *A. ilicifolia* populations.

**Figure 4:**
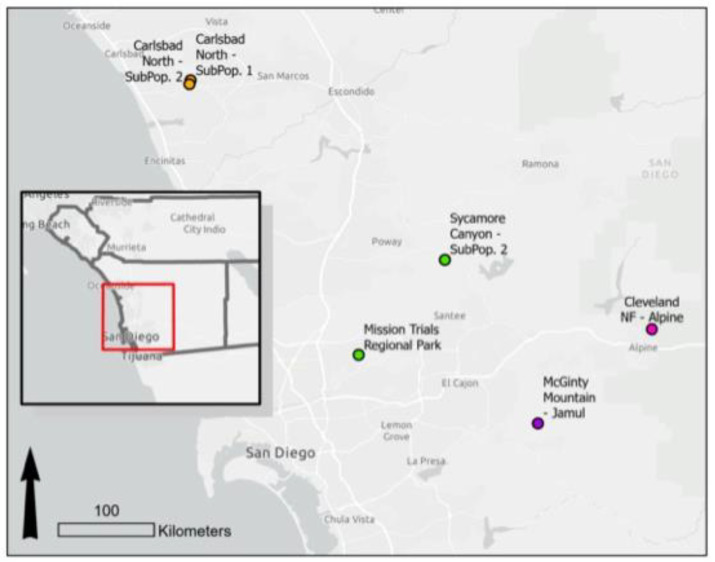
Map of the *Acanthomintha ilicifolia* populations represented in the common garden experiment. The colors of the population points represent the genetic clusters identified via a previous genetic analysis of this species [30], see Table 2.

The non-reproductive measures of plant production measured (biomass, height, and width) did not show predictable patterns along the regional rainfall gradient, but there were striking differences among populations. For instance, the Carlsbad subpopulations, located within a kilometer of each other, differed drastically in aboveground biomass and seed production. The most idiosyncratic population was the McGinty Mountain population, which responded more favorably to the high-irrigation treatment in terms of germination, flower production, and biomass. In contrast, the Alpine population, McGinty Mountain’s most similar neighbor in terms of geography (Figure 4), elevation, and rainfall (Table 2), did not show strong differences in response to aboveground allocation between treatments, and produced higher-quality, more viable seed under the low-water treatment. Past genomic work has demonstrated that, while only a few miles apart, these populations are from different genetic clusters [23]. While the belowground biomass was not evaluated in the present study, we acknowledge that the belowground allocation would likely be affected by the manipulation of belowground resource availability (e.g., water), as has been seen in prior studies on another southern California native, *Artemisia californica*, in which experimental drought stress increased the root-to-shoot ratio across a variety of treatments [27].

Maternal effects were not directly measured in the present study but are known to have important effects in common garden experiments [28] and on seed-based traits in particular [29]. While it is unclear how many maternal plants were represented in each population by treatment group at the end of the study, an effort was made to ensure that the seeds from each “stem” provided for seed processing were evenly distributed across the experiment. Based on the biology of the species, stems were likely roughly equivalent to maternal lines in this experiment, but future studies should endeavor to track and measure the maternal effect directly.

**Table 2:** The *Acanthomintha ilicifolia* populations represented in the common garden experiment.

Population Name	EO ^3^ (DeWoody et al. [22])	Genetic Cluster (Milano et al. [23])	Lat	Long	Elevation (m)	Wild Seed Collected	Mean Annual Rainfall (mm) ^1^	CV Ann. Precip. ^2^
Carlsbad1 (C1)	EO70A	Orange	33.14	−117.26	53	685	305	0.41
Carlsbad2 (C2)	EO70B	Orange	33.13	−117.26	53	2757	305	0.41
Sycamore Canyon (SC)	EO32-2	Green/mixed	32.93	−116.98	341	975	383	0.32
Mission Trails (MT)	EO33	Green	32.83	−117.07	153	588	312	0.40
McGinty Mountain (MM)	EO87l	Purple	32.75	−116.87	655	1369	363	0.34
Alpine (ALP)	EO75	Pink	32.86	−116.74	770	1705	504	0.25

^1^ 30 year normal annual rainfall values extracted from the Prism dataset 1990–2020 at 800 m resolution. ^2^ Coefficient of variation calculated from annual rainfall values extracted from the PRISM dataset 1980–2020 at 4 km resolution. ^3^ An elemental occurrence (EO) is an area of land in which a species or natural community is, or was, present. These numbers were assigned by the California Natural Diversity Database (CNDDB).

### 3.2. Enhanced Reproductive Performance in Low-Irrigation Treatment

Plants in the low-irrigation treatment tended to produce more seeds and higher-quality seeds, as measured via ex situ germination trials of the seed generated. It is unclear how the water availability in the experiment compares with natural responses to water resources, especially in the idiosyncratic clay soils that define *A. ilicifolia*’s range, but the lack of a clearly negative response in the reproductive output to what we believe is mild water stress is a positive indication for the species, as it experiences drought throughout its range. A similar common garden approach evaluating the watering treatment effects on 18 populations of *Lupinus angustifolius* in Spain found similar patterns, wherein low watering treatments produced plants with larger, higher-quality seeds, and the strength of this treatment effect varied among spatial, climatic gradients [26]. Further, our study aligns with findings across multiple species showing that plants increased their allocation to reproductive structures when grown under low-water conditions in a common garden setting (i.e., drought) [15,30].

Differing responses to water treatments across populations, including the trend toward an increased reproductive performance, suggest phenotypic plasticity (or epigenetic factors) influencing *A. ilicifolia*. Such plasticity may aid in short-term adaptation for climate change [7]. In the longer term, further study is needed to evaluate whether multiple generations of water stress would result in a qualitative trend of increased seed production in *A. ilicifolia* populations through epigenetic or biochemical effects, as has been seen in model systems [31], or selection for more fecund plants. Christmas et al. [7] note that competition from incoming species may be an ecological constraint to this adaptive potential. *A. ilicifolia* is threatened by invasive grasses, and previous work has indicated that seed production is negatively impacted by competition [32]. Further, we also observed that plants under the lower-watering treatment also flowered earlier than plants under the high-watering treatment. Phenological shifts in response to climate change can have negative consequences for the availability of pollinators [33] and the demographic dynamics of plant populations [34]. 

### 3.3. Conservation Strategy Implications of Results

For many conservation strategies, seed is required, to maintain or augment populations, or establish new populations within the existing range, or extend it. This study raises several interesting considerations for the strategy of maintaining a robust ex situ seed bank for *A. ilicifolia*. The results demonstrating the variety of responses to resource availability across populations emphasize the importance of conserving seed from across a species range to help preserve a variety of plant genotypes that may respond differently to climate change. The effect of watering treatments on plant performance and seed viability also indicates the importance of collecting seed in multiple years. In alpine species, seed collected in drier, warmer years has been shown to have a longer ex situ viability than seed produced in cooler, wetter years [35]. For annual species, the climate of the collection season may also influence the genetic makeup of the plants represented, especially following cases of severe drought [36].

Wild seed maintained a viability of >90% when stored at room temperature for seven years. This species appears to be extremely durable ex situ, which is good news for the effectiveness of seed banking as a safeguard against population extirpation. We do caution, however, that the conditions under which these seeds were stored are not ideal; it is always advised to store known “orthodox” seeds at −18 °C after seeds have been dried to a constant relative humidity for the best ex situ viability results [37]. It is difficult to know if the lack of appropriate conditions here altered the results for the seeds resulting from the common garden experiment, although we believe that the very high viability in the original wild collected seed helps assuage that concern.

Beyond long-term storage, our research also speaks to the curation and use of ex situ seed collections. Seed augmentations for restoration, or to replenish collections for this species, may be more successful when growing the plants under some stress (e.g., a low-water environment) to produce a higher quantity and quality of seed, an idea that warrants further research. Our observation of a much lower viability in seed produced from common garden treatments compared to wild collected seed stored in the same manner for the same amount of time indicates that pollinator limitation or environmental conditions less favorable to high-quality seed production may have reduced the seed set in the ex situ common garden. However, as all populations and treatments experienced common conditions, we do not believe that this lower production impacted the comparison results of the study. We recommend the hand pollination of this species for future experiments in an ex situ setting to prevent such a large viability loss, especially for those seeking to bulk seed for conservation translocations.

## 4. Materials and Methods

### 4.1. Population Selection

For this study, we selected six *A. ilicifolia* populations that would maximize the contrast across the species range in terms of proximity to the coast, elevation, and annual rainfall (Table 2). Two sub-populations from Carlsbad were selected to examine the intrapopulation variability across different microsites. The populations included in the common garden were later found to include four of the five genetic clusters identified via a regional analysis of 24 *A. ilicifolia* populations [23]. For each source population location, we extracted the 30 year normal annual rainfall (mm) and monthly historical rainfall from 1980 to 2020 from the PRISM climate explorer at 800 m and km resolution, respectively [38] (Table 2).

### 4.2. Common Garden Study

In 2012, the Center for Natural Lands Management collected stems with attached spiny nodes containing seeds from nine wild *A. ilicifolia* populations and provided material to the San Diego Zoo Wildlife Alliance. To access the seed, we broke open nodes with a rubber bung or a block of wood to release seeds, a process yielding between 170 seeds and 2757 per population (Table 2). CalFIRE’s L.A. Moran Reforestation Center performed X-ray analysis to determine whether the filled content of seed in each population sample was less than the 50-seed threshold. After X-ray analysis, three populations (La Costa Greens, Sycamore Canyon subpopulation 1, and Manchester) did not meet the filled seed threshold and were eliminated from the study. The common garden study began in February 2013 using six populations and two watering treatments (high and low) to observe the response of populations to variation in water availability. There were 55 pots per population per water treatment (660 total), which were then sown with 5 seeds per pot. The pots contained a substrate of 3:1 Sunshine#3 potting mix to washed sand in Anderson plant bands AB58 (5″ wide by 8″ tall). The potting mix was a fine soilless mix, which, when combined with sand, had a water-holding capacity similar to that of the native clay soil, and matched other garden studies conducted on the species [33].

**Figure 5:**
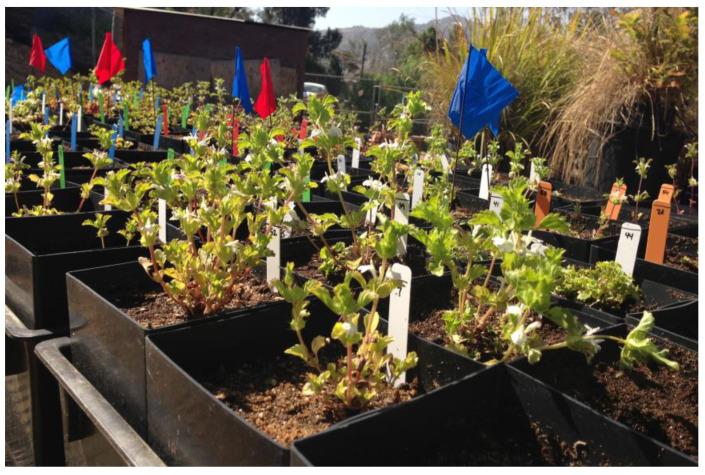
San Diego thornmint plants in the common garden experiment. Label markers enabled the tracking of individual plant flowering and reproduction, and flags indicated watering treatments.

The plants were grown in full sun on a single potting bench in the fenced horticultural growing area at the San Diego Zoo Safari Park (Escondido, CA, USA; see Figure 5) located within the species’ natural range. The experiment consisted of 37 trays, with nine pots per tray, and both the pots within the trays, and the tray location on the potting bench, were randomized.

During the germination phase, we watered the high-water pots deeply with 500 mL water biweekly and the low-water treatment with 500 mL water monthly, while keeping the soil moist with mist in both treatments between waterings. The mean annual precipitation was not considered for the nursery-grown plants due to container plants drying out more than plants in the ground and the variance in the mean precipitation across the range of the species. Rather than mimic natural precipitation levels, the goal was to provide common conditions and two distinct watering treatments. The number of germinants per pot was recorded each weekday for four weeks, and then thinned to one plant per pot. Each pot did not necessary represent the progeny of a separate maternal plant, because the wild seed was not separated by maternal line upon collection. After thinning, plants received 1000 mL per pot every two weeks (high treatment) or every four weeks (low treatment). The choice of 1000 mL per pot was not based on precipitation; rather, 1000 mL was enough to drip through the bottom of the pot, ensuring that the entire root system received water. The rate of high-water treatment being twice as frequent as that of the low-water treatment was likely to be different enough that variances in source population responses might be observed. However, we observed water stress in the growing plants, which prompted an increase in the watering frequency for both treatments, to 1000 mL weekly in the high-water treatment and 1000 mL biweekly in the low-water treatment.

Monitoring continued five days a week, with the recording of the date when each plant produced its first flower and the date of senescence. For the cross-pollination of the experimental plants, we relied on ambient pollinator activity in the well-vegetated area of the Safari Park, which has been successful at generating ample fertilization in numerous seed bulking and propagation trials in the vicinity. After senescence, we harvested plants and measured the following attributes: height, as the distance from the potting soil to the tallest vertical point on the plant; width, as the widest distance across the plant when viewed from the top down; the number of inflorescence whorls per individual; and the F1 seed quantity. As a measure of the aboveground biomass, we then dried all the non-seed aboveground material from each pot in a drying oven until the weight was constant. An attempt to collect the root biomass was abandoned due to difficulty in separating the fine roots from the soil.

The F1 seeds collected at harvest from the common garden experiment were stored under the ambient conditions of the seedbank from 2013 to 2019 in stable ~20–22 °C temperatures year-round, in sealed vials. The humidity within the seedbank was ~30–50%. However, the eRH (estimated relative humidity) of the seed within the vials during this period is unknown as, though sealed, it was not in flux.

### 4.3. Germination Protocols

For the germination tests, we plated 50–100 seeds (10 seeds per plate) for each of the different seed lots (wild and F1) involved in the study. The seeds were treated with a 1% bleach solution for 10 min to minimize contamination by mold, and then imbibed in R.O. water for 24 h prior to plating on a 0.5% agar medium. We labeled plate lids with the experimental treatment number and repetition number for data collection. We used ethanol to clean fine-tipped forceps while plating and when checking tests. The germination tests were placed into a germination chamber with an alternating baseline photoperiod day/night cycle of an 11 h day at 22 °C, and a 13 h night at 10 °C. We monitored tests and recorded the number of germinants every other day in the initial stages of the trials, then weekly as the seed germination rate plateaued. A seed was scored as germinated once the radicle emerged, and we removed germinants from the plate immediately to avoid the contamination of the remaining seeds.

### 4.4. Statistical Methods

#### 4.4.1. The Effect of Population and Watering Treatment on Plant Growth and Reproduction

We used generalized linear mixed-effect models to evaluate the interactive effects of the source population, the source population’s average annual rainfall, and the irrigation treatment on aspects of the aboveground growth, survivorship, and reproduction of *A. ilicifolia*. We evaluated the effect of the source population on the plant performance directly as a fixed effect in one series of models, and the effect of the average annual rainfall at the source population with the population as a random effect in a separate series of models. For the models evaluating the interactive effects of the source population and watering treatment, we specified the common garden bench position (east or west) as the random effect. For the models evaluating the interactive effects of the annual precipitation and the treatment as fixed effects, we specified the source population as a random effect. For binary dependent variables (germination, survivorship, and the presence of seed and flowers at harvest), we used a binomial error distribution; for count data (the total seed number across all plants in a treatment), we used a Poisson error distribution; and for all other continuous dependent variables (biomass, height, width, flower number, days to flower, seed number per plant and per unit biomass), we used a Gaussian error distribution. We natural-log-transformed the dependent variable seed number to meet the distributional assumptions of normality of errors. The models evaluating the average number of seed and inflorescences produced per plant included only plants that produced at least one flower or fruit, respectively. For each dependent variable, we present the Wald Type II test of fixed effects for the simplest model that did not improve the model AIC values by more than two points (Table A1 and Table A2). We used post hoc linear model contrasts to interpret the significance of the difference between high- and low-watering treatments within populations. Generalized linear models were implemented in the lme4 package in R [39].

#### 4.4.2. The Effect of Watering Treatments on Ex Situ Seed Viability

We statistically examined differences in ex situ germination rates among generations, populations, and treatments using cox proportional hazard regression models, a time-to-event method accounting for the number of germinants at each time point monitored. We fit one model with a generation-by-population interaction to test whether the seed produced from the experiment differed from the wild collected seed stored the same way for the same amount of time. We fit a second model with the treatment-by-population interaction evaluating the effect of the irrigation on the seed viability. Previous reviews of germination data analysis have advocated the use of Cox proportional hazard models in cases where researchers are contrasting the effect of treatments on germination outcomes [40]. We used the R package survival and function coxaph to implement the analysis [41].

#### 4.4.3. The Effect of Source Population Climate and Geographic on ex Situ Reproductive Output

To evaluate the ecological importance of the common garden and viability testing results at the population level, we calculated two aggregate measures of the reproductive output for the common garden treatment groups: the total seeds produced, summed over all plants in the treatment by population group, and the estimated viable seed produced (the total number of seeds produced multiplied by the population-by-treatment specific ex situ viability). We then used linear mixed models to test the interaction effect of the irrigation treatment by the source population’s average annual precipitation, elevation, latitude, and longitude on the following dependent variables: the total seeds produced, the ex situ viability, and the estimated viable seed produced. For each model, the population was specified as a random effect, and we used a Wald type II test of fixed effects to evaluate the significance of the fixed effects. We interpreted the simplest model that did not improve the model AIC values by more than two points.

## 5. Conclusions

Through our common garden approach, we learned more about how a rare species with a climatically heterogenous, but edaphically restricted, range responds phenotypically to differences in water availability. A previous report [22] exploring plants within just one of these watering treatments supported the hypothesis that genetic differences between populations exist and, thus, the differences seen across the species’ range are not solely due to plasticity expressed across environmental variability. However, adding the high- and low-watering treatments allowed us to identify differences in plasticity as a type of intraspecific variation. In particular, the added knowledge that water limitation may positively impact seed production and seed viability has implications for our understanding of the demography of *Acathomintha ilicifolia* under different climate scenarios, and merits further evaluation in situ.

Our results, in tandem with other work on the species [22,23], suggest that, across the range of the species, there is both adaptive genetic potential and phenotypic plasticity along the gradients of resource availability. As an edaphic endemic, the species’ migration potential is limited by the clay soil availability. Thus, using the framework set forth by Christmas et al. [7], we support the belief that the species conservation priority lies in the protection of genetic refugia and remnant populations. Specifically, we recommend land management strategies in key populations of each recognized genetic cluster [23]. Assisted migration would need to be explored if there are indicators that the adaptive capacity has been reached.

## Figures and Tables

**Table 1:** Common garden experiment results comparison for the plants’ aboveground and reproductive attributes after harvest according to population and irrigation treatment.

Population	Irrigation Treatment	Sample Size at Harvest	Biomass (g)		Height (cm)		Width (cm)		Floral Nodes		Seeds Produced per Plant	
			Mean	SE	Mean	SE	Mean	SE	Mean	SE	Mean	SE
Carlsbad 1	High	43	0.74	0.04	20.2	1.04	13.7	0.7	26.0	1.7	2.3	0.5
	Low	49	0.83	0.03	21.6	0.98	14.9	0.5	32.2	1.7	22.5	4.7
Carlsbad 2	High	49	0.42	0.05	12.6	0.76	9.8	0.7	14.4	1.6	6.6	1.1
	Low	47	0.57	0.03	14.0	0.62	13.0	0.5	22.4	1.3	17.3	4.7
Alpine	High	44	0.61	0.03	11.4	0.44	10.7	0.4	16.7	1.0	115.2	17.5
	Low	41	0.63	0.03	11.1	0.42	9.61	0.3	19.1	0.8	202.8	25.4
Mission Trails	High	44	0.59	0.05	14.0	0.60	9.8	0.5	19.6	1.7	14.3	5.7
	Low	43	0.76	0.04	14.9	0.52	11.2	0.4	26.6	1.4	56.5	10.3
McGinty Mountain	High	44	1.05	0.07	17.9	0.80	12.9	0.8	25.0	1.6	32.3	9.8
	Low	30	0.42	0.03	13.3	0.78	7.7	0.4	10.6	0.7	28.4	8.9
Sycamore Canyon	High	45	0.64	0.04	15.5	0.71	13.4	0.7	16.9	0.9	39.0	9.9
	Low	45	0.76	0.03	13.9	0.50	14.4	0.4	20.8	1.1	90.5	15.6

## Data Availability

Common garden datasets used for the manuscript were uploaded to the DRYAD repository (https://doi.org/10.5061/dryad.q573n5tpm).

## Appendix A

**Table A1:** The results of Wald type II chi-squared (*X*^2^) tests of fixed effects for generalized linear mixed-effect models evaluating the interactive effects of the source population and the irrigation treatment on aspects of San Diego thornmint growth and reproduction in the common garden experiment. For each dependent variable, we present the terms of the best model resulting from stepwise AIC model selection. Trt × pop = treatment-by-population interaction.

Dependent Variable	Model Terms	X^2^	df	*p*
Biomass at harvest	Treatment	0.1	1	0.807
	Population	12.2	5	<0.001
	Trt × pop	19.1	5	<0.001
Height at harvest	Treatment	0.5	1	0.484
	Population	175.0	5	<0.001
	Trt × pop	19.8	5	0.001
Width at harvest	Treatment	0.5	1	0.475
	Population	74.0	5	<0.001
	Trt × pop	43.1	5	<0.001
Number of Floral nodes	Treatment	9.2	1	0.002
	Population	86.7	5	<0.001
	Trt × pop	65.0	5	<0.001
Days to flower	Treatment	31.0	1	<0.001
	Population	41.2	5	<0.001
	Trt × pop	3.8	5	0.002
Number of Seed produced	Treatment	22.8	1	<0.001
	Population	165.5	5	<0.001
	Trt × pop	13.0	5	0.024
Number of Seed/biomass	Treatment	24.0	1	<0.001
	Population	199.8	5	<0.001
	Trt × pop	14.5	5	0.013
% Germination	Treatment	66.5	1	<0.001
	Population	41.4	5	<0.001
	Trt × pop	35.2	5	<0.001
% Survival	Treatment	3.1	1	0.651
	Population	3.3	5	0.08
	Trt × pop	15.3	5	0.009
% Plants producing seeds	Treatment	6.1	5	0.014
	Population	21.7	1	<0.001

**Table A2:** The results of the Wald type II chi-squared (X^2^) tests of fixed effects for linear mixed-effect models evaluating the interactive effects of the source population and the annual rainfall (mm) at the source population on aspects of San Diego thornmint growth and reproduction in the common garden experiment. For each dependent variable, we present the terms of the best model resulting from stepwise AIC model selection. Trt × ann. rain = treatment-by-annual-rainfall-at-wild-population interaction effect.

Dependent Variable	Model Terms	X^2^	df	*p*
Biomass at harvest	None retained	-	-	-
Height at harvest	None retained	-	-	-
Width at harvest	None retained	-	-	-
Number of floral nodes	Treatment	6.5	1	0.011
Days to flower	Treatment	30.3	1	<0.001
Number of Seed produced	Treatment	23.8	1	<0.001
	Ann. rain wild pop	74.4	1	<0.001
	Trt × ann. rain	8.2	1	0.004
Number of Seed/biomass	Treatment	24.5	1	<0.001
	Ann. rain wild pop	65.7	1	<0.001
	Trt × ann. rain	13.4	1	<0.001
% Germination	Treatment	40.3	1	<0.001
	Ann. rain wild pop	0.5	1	0.49
	Trt × ann. rain	16.3	1	<0.001
% Survival	None retained	-	-	-
% Plants producing seed	Treatment	5.9	1	0.024
	Ann. rain wild pop	5.1	1	0.016

**Table A3:** The results of the ex situ germination testing of seeds stored at the San Diego Zoo Botanical Conservation Center in sealed vials at room temperature for seven years after the completion of the common garden experiment. Wild seed indicates seed collected from the natural population used in the common garden experiment. CG indicates seed that was harvested from plants grown in the respective common garden treatments.

Population	Generation–Treatment	Ex Situ Germination (2019)
Carlsbad 1	Wild	100%
	CG-Hi	17%
	CG-Low	45%
Carlsbad 2	Wild	95%
	CG-Hi	25%
	CG-Low	42%
Alpine	Wild	N/A
	CG-Hi	25%
	CG-Low	55%
Mission Trails	Wild	85%
	CG-Hi	33%
	CG-Low	45%
McGinty Mountain	Wild	96%
	CG-Hi	49%
	CG-Low	54%
Sycamore	Wild	95%
	CG-Hi	48%
	CG-Low	75%

**Table A4:** The results of the Wald type II chi-squared (X^2^) tests of fixed effects for linear mixed-effect models evaluating the interactive effects of the source population and several climatic and geographic variables at the source population on the treatment-wide measures for reproductive output. For each dependent variable, we present the terms of the best model resulting from stepwise AIC model selection.

Dependent Variable	Model Terms	X^2^	df	*p*
	ANNUAL RAINFALL			
Total seeds	Ann. rain wild pop	52.1	1	<0.001
	Treatment	10.1	1	0.002
	Trt × ann. rain	6.4	1	0.011
Ex situ germ	Treatment	23.1	1	<0.001
Total viable seeds	Ann. rain wild pop	36.2	1	<0.001
	Treatment	12.9	1	<0.001
	Trt × ann. rain	13	1	<0.001
	ELEVATION			
Total seeds	Treatment	4.7	1	0.027
Ex situ germ	Elevation	23.1	1	<0.001
	Treatment	23.6	1	<0.001
Total viable seeds	Treatment	3.8	1	0.052
	LATITUDE			
Total seeds	Treatment	4.8	1	0.027
Ex situ germ	Treatment	23.1	1	<0.001
Total viable seeds	Treatment	3.8	1	0.052
	LONGITUDE			
Total seeds	Treatment	5.6	1	0.017
	Longitude	6.6	1	0.01
	Trt × longitude	1.8	1	0.181
Ex situ germ	Treatment	23.1	1	<0.001
Total viable seeds	Treatment	5.2	1	0.022
	Longitude	6.5	1	0.011
	Trt × longitude	2.9	1	0.091
